# Addressing the fetal alcohol spectrum disorder (FASD) ‘data gap’: Multi-method and multi-disciplinary public engagement to ascertain the acceptability and feasibility of establishing the first UK National linked database for FASD

**DOI:** 10.23889/ijpds.v8i2.2302

**Published:** 2023-09-14

**Authors:** Sarah Harding, Beverley Samways, Amy Dillon, Sandra Butcher, Andy Boyd, Raja Mukherjee, Penny Cook, Cheryl McQuire

**Affiliations:** 1 School for Policy Studies, University of Bristol, Bristol, United Kingdom; 2 NIHR Biomedical Research Centre, University of Bristol, Bristol, United Kingdom; 3 National Organisation for FASD, Hertfordshire, United Kingdom; 4 UK Longitudinal Linkage Collaboration, University of Bristol, Bristol, United Kingdom; 5 Foetal Alcohol Spectrum Disorder Service, Surrey and Borders Partnership NHS Foundation Trust, Surrey, United Kingdom; 6 School of Health and Society, University of Salford, Salford, United Kingdom; 7 Centre for Public Health, University of Bristol, Bristol, United Kingdom

## Objectives

To conduct public engagement work to establish the views of key stakeholders on the feasibility, acceptability, key purposes, and data structure of the first national linked longitudinal research database for fetal alcohol spectrum disorder (FASD) in the UK.

## Methods

We conducted stakeholder mapping and identified contacts through collaborator networks and online searches. We consulted with stakeholders using a range of formats, including: online focus groups (one for adults with FASD [and their supporters] N=5; one for guardians of people with FASD N=7), 1:1/small-team video calls and email communication with clinicians, data providers, policy-makers, data-governance experts, third-sector representatives and researchers [N=~85]), and one hybrid workshop (N=16 [N=10 in-person, 6 online attendees; 15 clinical and one third-sector]). Consultations included discussions on data sharing, harmonisation, perceived benefits, and challenges of a national linked database for FASD. We analysed consultation transcripts and notes using thematic analysis.

## Results

Our tailored, multi-method approach resulted in high levels of engagement with diverse stakeholders. Overall, stakeholders expressed strong support for a national linked database for FASD. For people living with FASD and third-sector representatives, the main perceived benefit was the potential for increased understanding, awareness, and support for FASD. Clinicians reported that a national database could provide new insights into FASD profiles, supporting more efficient diagnosis. Researchers highlighted potential for increased knowledge of FASD epidemiology and impacts. Policy-makers noted its clear alignment with contemporary FASD and data transformation policies. Common concerns were around privacy and data-sharing, particularly the tension between public good and disclosure risks if sample numbers were low. Clinicians expressed the importance of retaining space for clinical judgement and flexibility alongside the potential insights gained from quantitative analysis of data.

## Conclusion

Multi-method and multidisciplinary stakeholder engagement demonstrated the feasibility and acceptability of establishing the first UK national linked database for FASD. Perceived benefits and concerns varied by stakeholder group, demonstrating that flexible, diverse, embedded stakeholder collaboration will be essential as we seek to establish this database.

